# Psychological resilience and fear of disease progression among hospitalized patients with unruptured intracranial aneurysms scheduled for endovascular intervention: implications for nursing practice

**DOI:** 10.3389/fpubh.2026.1845542

**Published:** 2026-08-20

**Authors:** Xiaoxu Han, Wei Wang, Xin Xu, Lifang Zhu, Xiaofang Huang

**Affiliations:** Department of Nursing, The Second Affiliated Hospital Zhejiang University School of Medicine, Shangcheng, Hangzhou, China

**Keywords:** fear of disease progression, FOP, nursing care, psychological resilience, unruptured intracranial aneurysms

## Abstract

**Background:**

Hospitalized patients with unruptured intracranial aneurysms scheduled for endovascular intervention often experience uncertainty regarding disease progression during pre-treatment evaluation and treatment decision-making. Fear of disease progression arising from this uncertainty can have a substantial impact on patients' psychological health and quality of life. Psychological resilience, as a key psychological resource for coping with stress and uncertainty, has been shown to be associated with various psychological outcomes; however, its role in fear of disease progression among patients with unruptured intracranial aneurysms and the related implications for nursing care remain insufficiently explored.

**Methods:**

A cross-sectional, nurse-led observational study was conducted among hospitalized patients with unruptured intracranial aneurysms who were scheduled to undergo endovascular intervention at a tertiary teaching hospital in China. Psychological resilience was assessed using the Connor–Davidson Resilience Scale, and fear of disease progression was measured using the Fear of Progression Questionnaire–Short Form (FoP-Q-SF). Based on the established FoP-Q-SF cut-off score, participants were classified into a high FoP group (FoP-Q-SF ≥ 34) and a non-high FoP group (FoP-Q-SF <34). Multivariable linear regression analyses were performed to examine the associations between psychological resilience and overall FoP, as well as its subdimensions.

**Results:**

A total of 215 patients were included, of whom 71 (33.0%) were classified into the high FoP group and 144 (67.0%) into the non-high FoP group. Higher levels of psychological resilience were independently associated with lower overall FoP scores. Psychological resilience was also significantly associated with lower FoP–ER, whereas no significant association was observed with concerns related to functional impairment and social or family roles.

**Conclusion:**

Psychological resilience is inversely associated with fear of disease progression among hospitalized patients with unruptured intracranial aneurysms scheduled for endovascular intervention, particularly with emotional responses to disease-related uncertainty. These findings highlight the importance of assessing psychological resilience and fear of disease progression in routine nursing care. Future studies are warranted to evaluate whether nurse-led, emotionally focused supportive approaches may improve psychological outcomes in this population.

## 1 Introduction

Unruptured intracranial aneurysms (UIAs) are a common cerebrovascular disease. Previous epidemiological studies have shown that the prevalence of UIAs in the general population is about 3% (1, 2).With the wider availability and increased use of neuroimaging techniques, an increasing number of unruptured intracranial aneurysms are being detected incidentally in individuals without obvious symptoms (3). Because a substantial proportion of unruptured intracranial aneurysms (UIAs) are asymptomatic at the time of detection, clinical management is rarely a simple binary decision of “treat or not treat” (4). Instead, clinicians and patients typically need to weigh multiple management options—such as conservative surveillance, endovascular intervention, or microsurgical treatment—by considering aneurysm characteristics (e.g., location and size) as well as patient-specific risk profiles and expected benefits (5). Accordingly, regardless of the management strategy ultimately chosen, patients with UIAs often experience substantial uncertainty during disease evaluation and treatment decision-making, which may impose a considerable psychological burden.

Fear of disease progression (FoP) refers to the persistent worries and fears experienced by patients during the course of illness regarding the potential progression, recurrence, or deterioration of their disease and its possible consequences (6). Previous research has shown that FoP is common among patients with cancer and is associated with poorer psychosocial outcomes and reduced quality of life (7, 8). Beyond oncology, FoP has also been examined in other chronic disease populations, suggesting that it represents a broader psychological response to living with long-term illness (9). Unlike short-term and predictable disease courses, fear of disease progression often arises from uncertainty about the disease trajectory and patients' persistent concerns about their future health status (10, 11).

Psychological resilience is commonly conceptualized as an individual's capacity to maintain psychological stability, adopt positive coping strategies, and achieve adaptive adjustment when facing stress, uncertainty, and adversity (12). Psychological resilience has increasingly attracted attention in nursing and mental health research in recent years and is regarded as an important protective factor for patients' psychological adjustment and health outcomes (13). Previous studies in patients with cancer and various chronic diseases have shown that higher levels of psychological resilience are associated with lower anxiety and depressive symptoms and better quality of life, suggesting that resilience may buffer disease-related psychological responses (14). Previous studies have reported a negative association between psychological resilience and fear of disease progression, with higher resilience linked to lower levels of excessive worry about disease progression (15). Accordingly, patients with UIAs often experience substantial uncertainty during disease evaluation and treatment decision-making, which may impose a considerable psychological burden. However, research on the relationship between psychological resilience and fear of disease progression has largely focused on patients with cancer or other chronic illnesses (9), and evidence in patients with unruptured intracranial aneurysms remains limited.

While previous studies conducted in oncology and other chronic disease populations have suggested that psychological resilience is associated with lower levels of fear of disease progression and related psychological distress (15, 16), the conceptual proximity between these constructs raises questions regarding the added value of examining this relationship without considering disease-specific contexts.

Patients with unruptured intracranial aneurysms represent a clinically distinct population characterized by prolonged uncertainty, absence of overt symptoms, and ambiguity in treatment decision-making. Unlike conditions with a more predictable disease trajectory, individuals with UIAs often experience anticipatory concerns related to potential rupture and future health risks, despite the absence of immediate clinical deterioration.

In this context, examining the association between psychological resilience and fear of disease progression may provide insight into how general psychological resources relate to illness-specific emotional responses under conditions of uncertainty-dominant disease management.

Therefore, this study aimed to examine the association between psychological resilience and fear of disease progression among hospitalized patients with unruptured intracranial aneurysms scheduled for endovascular intervention, with a particular focus on its dimensional characteristics, to provide evidence for future nursing assessment and supportive care in this population.

## 2 Methods

### 2.1 Study design and participants

This study was a cross-sectional, nurse-led observational study conducted between March and October 2025 in the Department of Neurosurgery of a tertiary teaching hospital in Zhejiang Province, China. The study population consisted of hospitalized patients with unruptured intracranial aneurysms. A convenience sampling approach was used, and eligible patients were consecutively recruited. All questionnaires were administered during the pre-treatment assessment phase after hospital admission and prior to treatment initiation to ensure consistency in the timing of data collection and to reduce variability associated with different stages of the treatment process.

A total of 215 patients with unruptured intracranial aneurysms (UIAs) who were scheduled to undergo endovascular intervention were included. The inclusion criteria were as follows: (1) age ≥18 years; (2) a confirmed diagnosis of unruptured intracranial aneurysm based on imaging examinations; (3) admission to the hospital for treatment evaluation with a planned endovascular intervention; and (4) adequate cognitive and communication abilities to complete the questionnaires independently. The exclusion criteria included: (1) a history of severe psychiatric disorders (such as schizophrenia or bipolar disorder); (2) evident cognitive impairment or other conditions that precluded independent completion of the questionnaires; and (3) the presence of other severe neurological conditions that could substantially influence psychological status.

The questionnaires were administered by trained nursing staff within a fixed time window. Participants completed the questionnaires after receiving a full explanation of the study purpose and procedures, and the completed questionnaires were collected on site. The study protocol was approved by the hospital's Ethics Committee (approval number: 2024-1203). All participants provided written informed consent prior to participation.

The sample size was determined pragmatically based on recommendations for multivariable regression analyses. During the study design stage, approximately 15 candidate regression parameters derived from demographic, lifestyle, comorbidity, and aneurysm-related characteristics were anticipated for multivariable analyses. Following published recommendations for regression modeling, approximately 10–15 participants per regression parameter were considered appropriate (17). Accordingly, a minimum sample size of approximately 150–225 participants was considered adequate for study planning. The final sample included 215 participants, which was within this recommended range. This approach was used as a pragmatic guideline and does not itself guarantee stable regression estimates.

### 2.2 Measures

#### 2.2.1 Fear of disease progression

Fear of Progression Questionnaire–Short Form (FoP-Q-SF): Fear of disease progression was assessed using the Chinese version of the Fear of Progression Questionnaire–Short Form (FoP-Q-SF), which has demonstrated satisfactory reliability and validity in Chinese populations. The original Chinese validation study reported a Cronbach's α of 0.883 (18), and a subsequent validation study in Chinese cancer survivors further supported its reliability and validity (19). In the present study, Cronbach's α coefficients were 0.898 for the total scale, 0.834 for the FoP–ER subscale, and 0.816 for the FoP–FI subscale. The scale evaluates patients' worries and concerns related to disease progression and its potential impact on emotional wellbeing, daily functioning, and family life. Each item is rated on a 5-point Likert scale ranging from 1 (“never”) to 5 (“always”), with total scores ranging from 12 to 60. Higher scores indicate greater fear of disease progression. Consistent with previous studies using the FoP-Q-SF in patients with cancer and other chronic diseases, participants with a total score ≥34 were classified into the high FoP group (20). To our knowledge, this cut-off has not been specifically validated in patients with unruptured intracranial aneurysms; therefore, it was pragmatically adopted from the broader FoP literature for descriptive group comparisons. To preserve statistical information and avoid potential loss of statistical power associated with dichotomisation, all multivariable regression analyses were performed using continuous FoP total and subdimension scores as outcome variables. Accordingly, the dichotomised classification was used only for descriptive analyses, whereas all multivariable regression analyses were performed using continuous FoP scores.

#### 2.2.2 Psychological resilience

Connor–Davidson Resilience Scale (CD-RISC-25): Psychological resilience was measured using the Chinese version of the 25-item Connor–Davidson Resilience Scale (CD-RISC-25), which has demonstrated good reliability and validity in Chinese populations (12). The scale assesses an individual's capacity to cope with stress and adversity. Each item is rated on a 5-point Likert scale ranging from 0 (“not true at all”) to 4 (“true nearly all the time”), with higher scores indicating greater psychological resilience. Consistent with the commonly used three-factor structure in Chinese populations, resilience was assessed across three dimensions: tenacity, strength, and optimism (21). Mean scores were calculated for each dimension. In the present study, the CD-RISC-25 demonstrated excellent internal consistency, with a Cronbach's α of 0.951.

### 2.3 Covariates

Sociodemographic and clinical variables were collected using a structured questionnaire, including age, sex, body mass index, employment status, educational level, marital status, living arrangement, smoking history, alcohol consumption history, hypertension, diabetes mellitus, hyperlipidaemia, multiple aneurysms, and aneurysm location. These variables were considered potential covariates based on prior literature and clinical relevance.

### 2.4 . Statistical analysis

Descriptive statistics were used to summarize participant characteristics. Continuous variables are presented as means and standard deviations, and categorical variables as frequencies and percentages. Multivariable linear regression analyses were performed to examine the associations between psychological resilience and fear of disease progression, with FoP measures treated as continuous outcomes. Regression coefficients (β) and 95% confidence intervals (CIs) were reported. Additional exploratory analyses were conducted using the three resilience dimensions (tenacity, strength, and optimism) as alternative exposure variables. To evaluate the robustness of the findings, sensitivity analyses were performed using extended multivariable models with additional adjustment for sociodemographic, comorbidity, and aneurysm-related covariates. Statistical significance was defined as a two-sided *p* value <0.05. All statistical analyses were performed using *R* (version 4.4.2) and SPSS (version 26.0). Covariates included in the primary multivariable models were selected *a priori* based on previous literature and clinical relevance rather than statistical significance in univariable analyses. The primary multivariable models were adjusted for age, sex, smoking history, drinking history, and aneurysm location. Additional sociodemographic, comorbidity, and aneurysm-related variables were evaluated in sensitivity analyses to assess the robustness of the findings. Model fit was assessed using *R*^2^ and adjusted *R*^2^ statistics. Multicollinearity was evaluated using variance inflation factors (VIFs).

## 3 Results

### 3.1 Baseline characteristics and FoP levels by FoP risk group

A total of 215 participants were included in the analysis, comprising 144 patients in the non-high FoP group and 71 patients in the high FoP group. Baseline characteristics are presented in Table 1. Compared with the non-high FoP group, participants in the high FoP group had a lower prevalence of drinking history (18.3 vs. 33.3%, *P* = 0.022) and differed significantly in aneurysm location (*P* = 0.004). No significant differences were observed between the two groups with respect to age, sex, occupation, education level, marital status, smoking history, anxiety/sleep disorder, hypertension, diabetes mellitus, hyperlipidaemia, or aneurysm multiplicity (all *P* > 0.05).

**Table 1:** Comparison of baseline characteristics between the high and non-high FoP groups.

Variable	TotalP (*n* = 215)	Non-high FoPP (*n* = 144)	High FoP (*n* = 71)	Statistic	*P* value
Demographic characteristics					
Age (years)	58.61 ± 12.28	58.25 ± 12.66	59.34 ± 11.47	*t* = −0.632	0.528
Height (cm)	161.72 ± 7.63	162.26 ± 7.48	160.63 ± 7.88	*t* = 1.444	0.151
Weight (kg)	61.97 ± 10.35	62.24 ± 10.45	61.41 ± 10.15	*t* = 0.558	0.578
Body mass index (kg/m^2^)	23.58 ± 3.02	23.52 ± 2.97	23.71 ± 3.12	*t* = −0.421	0.674
Male sex, *n* (%)	70 (32.6)	51 (35.4)	19 (26.8)	χ^2^ = 1.623	0.203
Occupation, *n* (%)				χ^2^ = 0.652	0.884
Farming	62 (28.8%)	41 (28.5%)	21 (29.6%)		
Retired	79 (36.7%)	51 (35.4%)	28 (39.4%)		
Employed	65 (30.2%)	46 (31.9%)	19 (26.8%)		
Unemployed	9 (4.2%)	6 (4.2%)	3 (4.2%)		
Education level, *n* (%)				χ^2^ = 1.574	0.665
Primary school or below	75 (34.9%)	47 (32.6%)	28 (39.4%)		
Junior high school	69 (32.1%)	48 (33.3%)	21 (29.6%)		
High school/technical secondary school	37 (17.2%)	24 (16.7%)	13 (18.3%)		
College or above	34 (15.8%)	25 (17.4%)	9 (12.7%)		
Marital status, *n* (%)				χ^2^ = 4.232	0.237
Married	202 (94.0%)	137 (95.1%)	65 (91.5%)		
Single	5 (2.3%)	3 (2.1%)	2 (2.8%)		
Divorced	2 (0.9%)	2 (1.4%)	0 (0.0%)		
Widowed	6 (2.8%)	2 (1.4%)	4 (5.6%)		
Lifestyle factors					
Smoking history, yes, *n* (%)	45 (20.9)	31 (21.5)	14 (19.7)	χ^2^ = 0.094	0.759
Drinking history, yes, n (%)	61 (28.4)	48 (33.3)	13 (18.3)	χ^2^ = 5.281	0.022
Comorbidities					
Anxiety/sleep disorder, yes, *n* (%)	10 (4.7)	7 (4.9)	3 (4.2)	χ^2^ = 0.043	0.835
Hypertension, yes, *n* (%)	125 (58.1)	81 (56.3)	44 (62.0)	χ^2^ = 0.640	0.424
Diabetes mellitus, yes, *n* (%)	24 (11.2)	15 (10.4)	9 (12.7)	χ^2^ = 0.245	0.621
Hyperlipidaemia, yes, *n* (%)	38 (17.7)	27 (18.8)	11 (15.5)	χ^2^ = 0.347	0.556
Aneurysm characteristics					
Multiple aneurysms, *n* (%)				χ^2^ = 0.050	0.823
Single aneurysm	161 (74.9)	109 (75.7)	52 (73.2)		
Multiple aneurysms	54 (25.1)	35 (24.3)	19 (26.8)		
Aneurysm location, n (%)				χ^2^ = 10.859	0.004
Anterior circulation	188 (87.4%)	123 (85.4%)	65 (91.5%)		
Posterior circulation	24 (11.2%)	21 (14.6%)	3 (4.2%)		
Both anterior and posterior circulation	3 (1.4%)	0 (0.0%)	3 (4.2%)		

Continuous variables are presented as mean ± standard deviation (SD), and categorical variables as n (%). Continuous variables were compared using independent-samples t tests and categorical variables using Pearson's χ^2^ tests (or Fisher's exact tests where appropriate). Participants were classified into the high FoP group (FoP total score ≥34) and the non-high FoP group (FoP total score <34).

### 3.2 Association between psychological resilience and fear of disease progression

The associations between psychological resilience and fear of disease progression are presented in Table 2. After adjustment for age, sex, smoking history, drinking history, and aneurysm location, higher psychological resilience was independently associated with lower FoP total scores (β = −0.071, 95% CI: −0.140 to −0.002, *P* = 0.045). None of the covariates, including age, sex, smoking history, drinking history, or aneurysm location, were significantly associated with FoP total scores in the adjusted model (all *P* > 0.05). The model explained 7.0% of the variance in FoP total scores (*R*^2^ = 0.070; adjusted *R*^2^ = 0.039).

**Table 2:** Multivariable linear regression for FoP total score.

Variable	β	95% CI	*p* value
Psychological resilience (total score)	−0.071	−0.140 ~−0.002	0.045
Age (years)	−0.036	−0.144 ~ 0.071	0.508
Sex (male)	−1.375	−5.166 ~ 2.416	0.475
Smoking history (yes)	2.071	−2.422 ~ 6.564	0.365
Drinking history (yes)	−3.247	−6.903 ~ 0.409	0.081
Posterior vs anterior circulation	−1.953	−6.191 ~ 2.286	0.365
Both anterior and posterior circulation	10.924	−0.337 ~ 22.185	0.057

β represents unstandardised regression coefficients. FoP total score was treated as a continuous outcome. The model included psychological resilience total score as the primary predictor and was adjusted for age, sex, smoking history, drinking history, and aneurysm location, with anterior circulation as the reference category. Model fit: *R*^2^ = 0.070, adjusted *R*^2^ = 0.039.

### 3.3 Associations between psychological resilience and FoP subdimensions

The associations between psychological resilience and the two FoP subdimensions are presented in Table 3. After adjustment for age, sex, smoking history, drinking history, and aneurysm location, higher psychological resilience was independently associated with lower FoP–ER scores (β = −0.044, 95% CI: −0.086 to −0.003, *P* = 0.036). None of the covariates were significantly associated with FoP–ER scores (all *P* > 0.05). The model explained 6.8% of the variance in FoP–ER scores (*R*^2^ = 0.068; adjusted *R*^2^ = 0.037).

**Table 3:** Multivariable linear regression analyses of the associations between psychological resilience and FoP subdimension scores.

Variable	β	95% CI	*p* value	*R* ^2^	Adjusted *R*^2^
**Dependent variable: FoP–ER**				0.068	0.037
Const	22.814	18.144 ~ 27.484	0.000		
Psychological resilience total score	−0.044	−0.086 ~−0.003	0.036		
Age	−0.016	−0.080 ~ 0.049	0.632		
Sex	−1.243	−3.515 ~ 1.029	0.282		
Smoking history	0.299	−2.393 ~ 2.992	0.827		
Drinking history	−1.311	−3.502 ~ 0.880	0.239		
Aneurysm location (Posterior circulation)	−0.821	−3.362 ~ 1.719	0.524		
Aneurysm location (Both anterior and posterior circulation)	5.435	−1.314 ~ 12.184	0.114		
**Dependent variable: FoP–FI**				0.072	0.040
Const	14.848	11.242 ~ 18.454	0.000		
Psychological resilience total score	−0.027	−0.059 ~ 0.005	0.103		
Age	−0.021	−0.070 ~ 0.029	0.417		
Sex	−0.132	−1.886 ~ 1.622	0.882		
Smoking history	1.772	−0.307 ~ 3.851	0.094		
Drinking history	−1.936	−3.627 ~−0.244	0.025		
Aneurysm location (Posterior circulation)	−1.131	−3.092 ~ 0.830	0.257		
Aneurysm location (Both anterior and posterior circulation)	5.489	0.278 ~ 10.700	0.039		

All regression models were ordinary least squares linear regression. Reference group for aneurysm location: anterior circulation. β: regression coefficient; 95% CI: 95% confidence interval; *P*: *P*-value; *R*^2^: coefficient of determination; Adjusted *R*^2^: adjusted coefficient of determination. FoP–ER: Fear of Progression–Emotional Response subdimension; FoP–FI: Fear of Progression–Functional Impairment subdimension. All VIF values were below 5, indicating no evidence of problematic multicollinearity

In contrast, psychological resilience was not significantly associated with FoP–FI scores after adjustment for the same covariates (β = −0.027, 95% CI: −0.059 to 0.005, *P* = 0.103). Drinking history was independently associated with lower FoP–FI scores (β = −1.936, 95% CI: −3.627 to −0.244, *P* = 0.025). In addition, compared with patients with aneurysms located in the anterior circulation, those with aneurysms involving both the anterior and posterior circulation had higher FoP–FI scores (β = 5.489, 95% CI: 0.278 to 10.700, *P* = 0.039). No significant associations were observed for the remaining covariates (all *P* > 0.05). The model explained 7.2% of the variance in FoP–FI scores (*R*^2^ = 0.072; adjusted *R*^2^ = 0.040).

## 4 Discussion

This study examined the association between psychological resilience and fear of disease progression among hospitalized patients with unruptured intracranial aneurysms scheduled for endovascular intervention. The findings showed that approximately one-third of patients were classified as having a high FoP group (FoP-Q-SF ≥ 34). After adjusting for sociodemographic and lifestyle factors, psychological resilience was inversely associated with overall FoP levels, with this association primarily observed in the emotional response domain (FoP–ER). Although the observed associations reached statistical significance, the magnitude of the effects was relatively modest, suggesting that psychological resilience represents one of several factors associated with fear of disease progression rather than its sole determinant.

Among hospitalized patients with unruptured intracranial aneurysms awaiting endovascular intervention, the inherent unpredictability of the disease and its potentially serious consequences may readily evoke concerns about future disease progression, which are often manifested as emotional responses such as fear, anxiety, and repetitive worry (22). In the present study, psychological resilience was primarily associated with FoP–ER, whereas no significant association was observed with FoP–FI. This finding appears theoretically plausible. Although psychological resilience and fear of disease progression are conceptually related constructs, as both involve emotional regulation and responses to stress and uncertainty, they are generally regarded as distinct psychological domains. Psychological resilience reflects a broader adaptive capacity for coping with adversity, whereas FoP represents illness-specific cognitive and emotional concerns regarding future disease deterioration and its consequences (23). Therefore, while some degree of conceptual overlap cannot be excluded, the observed association suggests that greater adaptive psychological resources are related to lower levels of emotional distress when patients are confronted with disease-related uncertainty, rather than simply reflecting redundancy between psychological constructs.

From a theoretical perspective, the association between psychological resilience and FoP may be understood through several related psychological mechanisms. First, resilience may be linked to patients' perceived control when facing illness-related uncertainty. Patients with greater resilience may be more able to maintain a sense of controllability and psychological stability when confronted with uncertain disease trajectories and treatment decisions, which may be associated with lower emotional responses to FoP. Second, intolerance of uncertainty may also be relevant in patients with UIAs, because the disease is often asymptomatic but potentially serious, and patients may experience persistent concerns about rupture risk, treatment outcomes, and future health status. In this context, resilience may not operate as an isolated protective factor, but rather as part of a broader psychological network involving perceived control, uncertainty appraisal, coping resources, social support, and self-management ability. Therefore, the present findings should be interpreted as evidence of an association between resilience and FoP-related emotional responses, rather than as evidence that resilience independently determines FoP.

Compared with FoP–ER, concerns related to FoP–FI are more closely tied to real-life role functioning, family responsibilities, and the availability of social support. These aspects are largely influenced by disease status, family structure, and broader social and environmental conditions, and may not be fully determined by individuals' internal psychological resilience. In addition, previous research has suggested that individuals' perceived sense of control in stressful situations may represent a key mechanism underlying psychological resilience, and is closely associated with FoP–ER, neuroendocrine stress responses, and overall psychological wellbeing (24). From this perspective, the observed association between psychological resilience and FoP–ER may indicate that these constructs are more closely related at the level of subjective emotional responses to disease-related uncertainty.

In addition, previous research (25) has shown that psychological resilience often does not operate in isolation, but works together with other psychological resources, such as patients' self-management abilities, to influence psychological outcomes. These findings suggest that psychological resilience may be more closely associated with the emotional response component of FoP than with concerns related to functional impairment and social/family concerns.

Previous studies adopting a psychosocial adjustment perspective have suggested that psychological resilience often works together with other psychological and behavioral resources, such as social support and self-management abilities, to influence patients' psychological outcomes (8). This perspective emphasizes that fear of disease progression is unlikely to be determined by a single psychological trait, but rather reflects the combined influence of multiple psychological and social factors. The findings observed in patients with unruptured intracranial aneurysms in the present study extend existing evidence largely derived from cancer and other chronic disease populations, and suggest that the association between psychological resilience and fear of disease progression may be observed across different disease contexts despite substantial differences in clinical characteristics and disease trajectories.

Sensitivity analyses showed that the direction of the association remained consistent after additional adjustment, although the association was attenuated and no longer reached statistical significance (Supplementary Table S1).

## 5 Implications for nursing practice

The findings of this study have important implications for clinical nursing practice. Approximately one-third of hospitalized patients with unruptured intracranial aneurysms awaiting endovascular intervention were classified as having high fear of disease progression. These findings support incorporating psychological resilience and fear of disease progression into routine pre-treatment nursing assessment to facilitate early identification of patients who may require additional psychological support.

Given that the association between psychological resilience and fear of disease progression was primarily observed for the emotional response dimension, nursing interventions may particularly focus on identifying patients with prominent emotional concerns. Such patients may benefit from individualized health education, emotional support, effective communication regarding disease and treatment, and referral for psychological counseling or multidisciplinary supportive care when appropriate.

Although the observed associations were statistically significant, their magnitude was modest. Therefore, psychological resilience should be considered one of several factors associated with fear of disease progression rather than the sole determinant, and future prospective studies are warranted to determine whether resilience-based nursing interventions result in clinically meaningful improvements in psychological outcomes.

Second, this study found that psychological resilience was primarily associated with the emotional response dimension of fear of disease progression, suggesting that clinical nurses may place greater emphasis on patients' emotional experiences and emotion regulation when implementing nursing interventions. In particular, for patients with more pronounced emotional responses related to fear of disease progression, as well as those with relatively lower levels of psychological resilience, nursing assessment and support may, in addition to routine care, focus more on their emotional experiences, stress responses, and subjective perceptions of disease-related uncertainty. Previous research from a psychosocial adjustment perspective has indicated that psychological resilience works together with other psychological or behavioral resources, such as social support and self-management abilities, to influence psychological outcomes. This highlights the importance of providing emotional support, health education, and facilitating patients' active coping to help them better manage disease-related uncertainty (8, 26). In contrast, for patients whose fear of disease progression is dominated by concerns related to functional impairment and social or family roles, their distress may be more strongly influenced by family responsibilities, levels of social support, and real-life contextual factors. During follow-up and health education, nurses may place greater emphasis on assessing patients' caregiving burden, recovery of role functioning, and availability of social support, encourage the involvement of family members in the care process, and, when appropriate, facilitate multidisciplinary collaboration and linkage to relevant social resources. These approaches may help support patients in better adapting to disease-related changes in daily life.

In addition, previous studies have suggested that psychological resilience often works together with other psychological or behavioral resources, such as social support and self-management abilities, to influence patients' psychological outcomes (8). Evidence from randomized trials suggests that interventions incorporating therapist guidance may achieve greater reductions in fear of progression/recurrence than providing self-help resources alone (27). Together, these findings, in conjunction with previous literature, support the potential value of incorporating psychological assessment and supportive care into routine nursing practice. Future intervention studies are needed to evaluate whether nurse-led psychological support strategies can improve psychological outcomes in this population.

## 6 Limitations

This study has several limitations. First, given the cross-sectional nature of this study, the directionality of the observed association cannot be determined. Although higher psychological resilience was associated with lower levels of fear of disease progression, reverse causality cannot be excluded. Longitudinal studies are needed to clarify the temporal relationship between psychological resilience and fear of disease progression.

Second, although we adjusted for a range of demographic, lifestyle, comorbidity, and aneurysm-related characteristics and confirmed the robustness of the findings through sensitivity analyses, the possibility of residual confounding remains. Important psychological and clinical factors, including illness perceptions, treatment decision-related factors, aneurysm size, estimated rupture risk, and validated assessments of anxiety and depressive symptoms, were not available in the present study. In addition, because assessments were conducted after hospital admission and before endovascular treatment, some participants' responses may have been influenced by hospitalization-related stress or concerns regarding the upcoming procedure. Although the FoP-Q-SF is designed to assess worries related to disease progression rather than procedural anxiety, the potential overlap between these psychological responses cannot be completely excluded.

Third, participants were recruited from a single tertiary center using convenience sampling and consisted exclusively of hospitalized patients scheduled for endovascular intervention. Hospitalization and anticipation of an upcoming procedure may have influenced psychological responses. Therefore, the findings may not be fully generalisable to patients managed conservatively, followed in outpatient settings, or undergoing microsurgical treatment.

Future multicentre longitudinal studies incorporating more comprehensive clinical and psychological assessments are warranted to further validate and extend the present findings.

## 7 Conclusions

This study explored the association between psychological resilience and fear of disease progression among hospitalized patients with unruptured intracranial aneurysms scheduled for endovascular intervention. The findings indicate that a substantial proportion of patients experience high levels of fear of disease progression, and that psychological resilience is inversely associated with overall fear of disease progression. This association was primarily observed in the emotional response dimension, whereas no significant association was found with concerns related to FoP-FI.

These findings suggest that psychological resilience may be related to lower levels of fear of disease progression, particularly within the emotional response dimension. No significant association was observed with concerns related to functional status or social and family roles. From a nursing perspective, early identification of patients with pronounced emotional responses and lower levels of psychological resilience may help inform risk stratification and guide the provision of targeted psychological support.

Overall, this study underscores the importance of incorporating psychological resilience and fear of disease progression into routine nursing assessment for patients with unruptured intracranial aneurysms. The findings also suggest that nurse-led, emotionally focused supportive interventions may represent a promising strategy for improving patients' psychological wellbeing and should be further evaluated in prospective intervention studies.

## Data Availability

The raw data supporting the conclusions of this article will be made available by the authors, without undue reservation.
