# Supplementary material for: Psychological resilience and fear of disease progression among hospitalized patients with unruptured intracranial aneurysms scheduled for endovascular intervention: implications for nursing practice
Source: Front Public Health. 2026 Aug 20;14:1845542. doi: 10.3389/fpubh.2026.1845542 (PMC13537977; doi:10.3389/fpubh.2026.1845542)
Supplement: Supplementary file 1 [file Table_1.docx]

**S1. Sensitivity analyses of the associations between psychological resilience and fear of disease progression**

| **Model ID** | **Outcome** | **Adjustment** | **β** | **95% CI** | **P value** | **R²** | **Adjusted R²** |
| --- | --- | --- | --- | --- | --- | --- | --- |
| S1 | FoP total score | Unadjusted | -0.074 | (-0.143, -0.005) | 0.036 | 0.020 | 0.016 |
| S2 | FoP total score | Primary model* | -0.071 | (-0.140, -0.002) | 0.045 | 0.070 | 0.039 |
| S3 | FoP total score | Fully adjusted* | -0.059 | (-0.129, 0.011) | 0.098 | 0.111 | 0.058 |

Table footnote: Primary model: adjusted for age, sex, smoking history, drinking history, and aneurysm location. *Fully adjusted model: additionally adjusted for education level, marital status, hypertension, diabetes mellitus, and hyperlipidaemia.
